# Additive genetic variation, but not temperature, influences warning signal expression in *Amata nigriceps* moths (Lepidoptera: Arctiinae)

**DOI:** 10.1002/ece3.9111

**Published:** 2022-07-17

**Authors:** Georgina E. Binns, Liisa Hämäläinen, Darrell J. Kemp, Hannah M. Rowland, Kate D. L. Umbers, Marie E. Herberstein

**Affiliations:** ^1^ School of Natural Sciences, 14 Eastern Road Macquarie University North Ryde New South Wales Australia; ^2^ Max Planck Institute for Chemical Ecology Hans Knöll Straße 8,Jena Germany; ^3^ School of Science Western Sydney University Penrith New South Wales Australia; ^4^ Hawkesbury Institute for the Environment Western Sydney University Penrith New South Wales Australia

**Keywords:** antipredator, aposematism, heritability, Lepidoptera, thermal melanism

## Abstract

Many aposematic species show variation in their color patterns even though selection by predators is expected to stabilize warning signals toward a common phenotype. Warning signal variability can be explained by trade‐offs with other functions of coloration, such as thermoregulation, that may constrain warning signal expression by favoring darker individuals. Here, we investigated the effect of temperature on warning signal expression in aposematic *Amata nigriceps* moths that vary in their black and orange wing patterns. We sampled moths from two flight seasons that differed in the environmental temperatures and also reared different families under controlled conditions at three different temperatures. Against our prediction that lower developmental temperatures would reduce the warning signal size of the adult moths, we found no effect of temperature on warning signal expression in either wild or laboratory‐reared moths. Instead, we found sex‐ and population‐level differences in wing patterns. Our rearing experiment indicated that ~70% of the variability in the trait is genetic but understanding what signaling and non‐signaling functions of wing coloration maintain the genetic variation requires further work. Our results emphasize the importance of considering both genetic and plastic components of warning signal expression when studying intraspecific variation in aposematic species.

## Graphical abstract

Warning signal variability can be explained by trade‐offs with other functions of colouration, such as thermoregulation, that may constrain warning signal expression by favouring darker individuals. Here, we investigated the effect of temperature on warning signal expression in aposematic *Amata nigriceps* moths that vary in their black and orange wing patterns. We found no effect of temperature on warning signal expression in either wild or laboratory‐reared moths, and instead, found sex‐ and population‐level differences in wing patterns, and high levels of trait heritability.

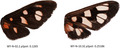

## 1 INTRODUCTION

The colors of animals have long been used to understand adaptation and fitness in natural environments (Bates, 1862; Cook & Saccheri, 2013; Cuthill et al., 2017). Research often identifies a single function of external appearances, but color patterns can experience multiple, often opposing, selection pressures (Cuthill et al., 2017; Ruxton et al., 2018). How organisms resolve these trade‐offs depends on the shape of the fitness curve resulting from the different selective forces and leads to the diversity of color patterns that we see in the natural world (Cuthill et al., 2017). Some of the most striking examples of these colorations are those of aposematic animals which signal their unprofitability to predators with bright and conspicuous warning signals (Poulton, 1890).

Variability in the warning signals of aposematic animals is a topic of repeated discussion (Briolat et al., 2018), because aposematism is most successful when the primary warning signal is consistent and recognizable throughout the population (Lindström et al., 2001; Rowland et al., 2010). We, therefore, expect stabilizing selection resulting in signal uniformity within populations (Endler, 1988; Mallet & Joron, 1999), but we see repeated examples of both within (e.g., Blount et al., 2012; Lindstedt et al., 2009; Nokelainen et al., 2012; Rojas & Endler, 2013) and between population (e.g., Fabricant et al., 2018; Maan & Cummings, 2008; Mochida, 2009), variability in warning signals. This variation can be genetically determined or plastic and result from a combination of biotic and abiotic factors that vary spatially and temporally (Briolat et al., 2018). These include heterogeneity among predators (Endler & Mappes, 2004; Nokelainen et al., 2014), composition and selection dynamics relative to co‐mimics (Mallet & Joron, 1999), and environmental conditions, such as temperature, rainfall, and resource availability, which can affect the physiology of signal expression (Blount et al., 2012; Fabricant et al., 2018; Lindstedt et al., 2009). Besides working as an antipredator defense, signals may also be important in intraspecific interactions, such as in mate choice or competition (Crothers & Cummings, 2015; Nokelainen et al., 2012), which adds another layer of complexity to the observed signal diversity.

Temperature is one of the most important abiotic selection pressures influencing warning signal expression. According to Gloger's rule, animal coloration is related to broad‐scale climatic gradients, and individuals are expected to be darker in warmer and more humid environments (Delhey, 2019; Gloger, 1833). However, recent work suggests that this relationship is mainly driven by humidity (Delhey, 2019), and in many cases, the effect of temperature is the opposite, consistent with the thermal melanism hypothesis (Clusella‐Trullas et al., 2007). This hypothesis states that species in colder climates should produce darker coloration that leads to higher body temperatures, which is expected to be particularly important for ectotherms (Clusella‐Trullas et al., 2007; Dalrymple et al., 2018). Indeed, the association between dark coloration and cold environments has been documented in many insects (e.g., Davis et al., 2005; Fabricant et al., 2018; Karl et al., 2009; Lewis, 1985; Rosa & Saastamoinen, 2020; Solensky & Larkin, 2003), and several studies have demonstrated that this results in fitness benefits (reviewed in Clusella‐Trullas et al., 2007, but see Umbers et al., 2012 for contrasting results). In aposematic species, this could create a trade‐off between thermoregulation and antipredator defense if increased melanization limits the amount of other pigments needed for the warning signal (Hegna et al., 2013; Lindstedt et al., 2009). Thermal melanism might, therefore, constrain warning signal expression, and the relative costs and benefits of melanin in different temperatures could maintain signal variation in aposematic species (Hegna et al., 2013, Lindstedt et al., 2009).

In addition to environmental factors, the heritability of traits is key to understanding warning signal variability. In many species, variation in warning signals results from a combination of genetic differences and plastic responses to the environment (e.g., Davis et al., 2005; Lindstedt et al., 2009). For example, the size of the warning signal (orange patch) in wood tiger moth larvae (*Arctia plantaginis*) is highly heritable, but signal size varies in response to temperature (Lindstedt et al., 2009) and predation pressure during development (Abondano Almeida et al., 2021). These plastic responses can be shaped by genotype‐environment interactions where families or populations show different reaction norms to the environmental conditions due to a genetic differentiation in developmental plasticity (Via & Lande, 1985). For example, melanism in many insects tends to have flatter reaction norms in tropical populations compared with temperate populations (Gibert et al., 1996; Roskam & Brakefeld, 1996). To what extent a genotype of a given organism can accommodate environmental gradients is central to our understanding of warning signal plasticity, and to our capacity to predict how species will respond to global climate change.

**FIGURE 1:**
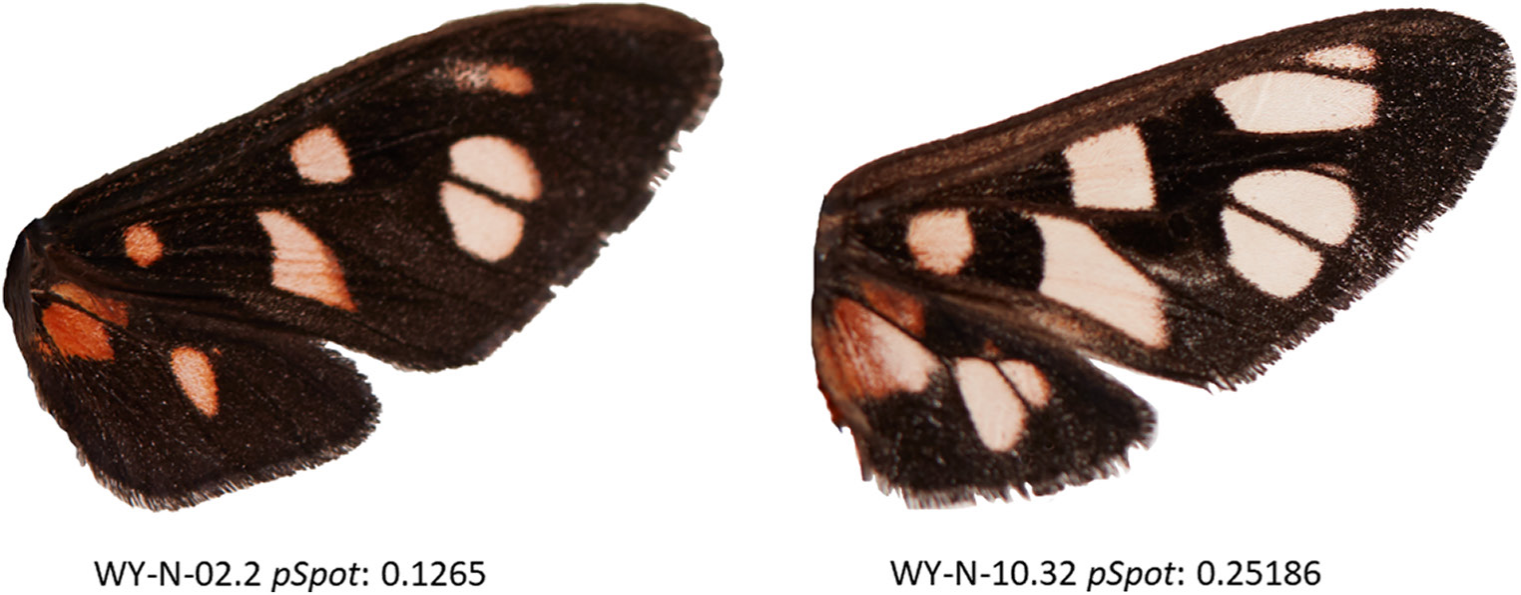
Examples of intraspecific variation in *Amata nigriceps* wing patterns. Both images are from the moths from the Wyoming population that were reared in the laboratory on the same diet. The proportion of orange is 0.13 in the wing on the left and 0.25 in the wing on the right

Here, we investigate environmental and genetic sources of variation for warning signal expression in the aposematic wasp moth, *Amata nigriceps*. The moths secrete defensive neck fluids, which appear to be unpalatable to bird predators and utilize pyrazines as an odor defense (Rothschild et al., 1984), and their warning coloration includes bright orange wing spots contrasted against a black background (Figure 1). The species is likely to be part of a mimicry complex consisting of several species in the *Amata* genus, but these mimetic relationships are still poorly studied. The coverage of orange on the wings has been shown to vary between 10 and 30% within and across *A. nigriceps* populations (Binns et al. in review). Previous work has demonstrated that avian predators can discriminate between 15% and 22% orange wing signals (Hämäläinen et al. unpublished), and selection from predators is expected to favor more conspicuous (more orange) warning signals (Speed, 2000), which makes this variation puzzling.

One possible mechanism for maintaining this signal diversity is variation in environmental temperatures. Although the pigment basis of the moth coloration is still unknown, darker coloration is expected to provide thermoregulation benefits, which could lead to smaller warning signals in lower temperatures (Clusella‐Trullas et al., 2007), but so far this has not been tested. Furthermore, we do not know if variation in *A*. nigriceps warning signals is plastic, driven by selection, or results from an interaction between both environmental and genetic components. This is crucial if we are to understand how quickly the species can adapt to rapidly changing environmental conditions. We compared warning signals in (i) wild moths collected from the same population during spring and summer emergence periods and (ii) in moths reared in the laboratory in three different temperatures (20, 24, and 28°C). We predicted that lower temperatures would extend larval development time and result in smaller orange patches in adult wings. We also report population‐level reaction norms and estimate heritability in warning signal traits. Finally, because much of the ecology of the species is still poorly understood, we provide important insights into the life history of the moths.

## 2 METHODS

### 2.1 Study species

The Australian red‐necked wasp moth *Amata nigriceps* (Lepidoptera: Syntomini) is a diurnal moth with conspicuous orange wing spots against a black background. The moths are commonly distributed along the east coast of Australia from Victoria, throughout New South Wales to Queensland, which ranges from temperate bushland to rainforest, to coastal and urban‐disturbed habitats. *Amata,* as a genus, is polyphagous, and the larvae feed on dead plant material (Common, 1990) but have been also raised successfully on rose petals (*Rosa sp*.), common dandelion leaves (*Taraxacum sp*.), bladder saltbush (*Atriplex vesicaria*), dead Eucalypt leaves (Common, 1990), and on artificial diet (pers. obvs.). The species is bivoltine and has two flying seasons, the first season in spring (from October to December), and the second season in late summer (from February to April, pers. obvs.). The moths overwinter as larvae between April and September. During the summer, the larval stage is approximately 2 months, and the pupal stage lasts for 10–20 days (see rearing experiment results for details).

### 2.2 Seasonal differences in a wild population

To investigate the effects of seasonal differences in environmental temperature on *A. nigriceps* warning signals, we captured 220 moths from Macquarie Park, Sydney (Wallumattagal Land: 33°46′25.77″ S 151°06′45.54″ E) during years 2017–2020 and quantified their warning signal size. Seventy‐eight adult moths were collected during the spring season from October to December (13 females and 65 males) and 142 moths during the late summer season from February to April (38 females and 104 males). To estimate seasonal differences in temperature during larval development, we calculated mean minimum and maximum temperatures two months prior to adult collection when the moths were in the larval and pupal stages. During 2017–2020, spring seasons (August–October) in Macquarie Park experienced mean temperatures of 10.8–20.3°C, and the summer season (December–February) temperatures averaged 18.2–27.1°C (http://www.bom.gov.au/climate/data). All collected moths were euthanized and stored in a − 30°C freezer. The moths collected between 2017 and 2019 (*n* = 83) were pinned and then photographed for wing color pattern analysis (using Nikon D90 camera, see details in Binns et al. in review). The moths collected in 2020 (*n* = 137) were used later for a chemical analysis, and instead of pinning, we carefully removed one forewing and hindwing from each individual, choosing the wings that were better preserved. The wings were then photographed, and images were processed as described below (image processing).

### 2.3 Rearing experiment

Twenty mating pairs were collected from October to November 2020 from three locations in New South Wales, Australia: Macquarie Park, Sydney (*n* = 3 mating pairs)—a mostly urban environment, with temperate eucalypt forest national park surrounds (Wallumattagal Land, 33°46′25.77″ S 151°06′45.54″ E); Wyoming, Central Coast (*n* = 13 mating pairs)—predominantly residential area surrounded by temperate‐subtropical rainforest natural reserves (Guringai Land, 33°24′23.39″ S 151°21′37.60″ E); and Tomaree Mountain, Shoal Bay (*n* = 4 mating pairs)—National Park mountainous coastal heathland elevating to 162 m (Worimi Land, 32°43′05.48″ S 152°10′58.58″ E). The sites had similar maximum daily temperatures, but Mt Tomaree had a higher mean rainfall during the larval season compared with the other two sites (see Supplementary material). After mating, females were moved to 250‐ml plastic containers and allowed to lay eggs on the inside surface of the container for 2 days. The females were then removed, and the eggs kept at room temperature (~22°C) and under a 12/12 h artificial light/dark cycle. Once larvae hatched (approximately 8 days after egg laying), they were allowed to feed on egg casings for 24 h before being supplied with fresh dandelion leaves that were collected from around Macquarie University Campus. After 10 days at room temperature (~22°C) in the laboratory (following rearing protocol; Lindstedt et al., 2009), 964 1st instar larvae from 17 different families (Macquarie: *n* = 184; Mt Tomaree: *n* = 286; Wyoming: *n* = 494, see Supplementary material for details about family representation) were randomly assigned into one of three temperature treatments and placed into temperature‐controlled growth chambers (Conviron® CMP6050). These chambers were set to a 16 L/8Dhr light cycle, mimicking the photoperiods experienced during Australian East coast summer, 65% humidity (average December humidity) and three temperature settings: (A) 20°C day/16°C night (*n* = 320 larvae), (B) 24°C day/20°C night (*n* = 323 larvae), and (C) 28°C day/24°C night (*n* = 321 larvae).

Larvae were kept in family groups of 20 in 250‐ml plastic containers (8 × 5.5 × 5.5 cm) covered with fabric fastened with elastic bands. The containers were sprayed with water, and fresh dandelion leaves were provided daily. Once a week, larvae were removed from containers for cleaning, dead larvae were removed, and mortality was recorded. Once the majority of larvae reached the second instar, larvae were moved to larger (500 ml) take‐away food containers (16.5 × 11.5 × 6.5 cm).

Larvae were allowed to pupate in the rearing containers and were then removed from the group‐rearing containers and placed in individual cylindrical collection containers (100 ml; 6.5 × 4 cm). We recorded the date of pupation and the total number of days as a larva and measured the pupal weight to three decimal places (using Sartorius Precision balance BP 150). In some cases, the pupae were damaged or partly eaten by the other larvae in the container (20°C: *n* = 12 pupae, 24°C: *n* = 3 pupae). These were included in the number of larvae that survived until pupation (see results), but we did not record other measures of them. Pupated individuals were returned to the growth chambers, and they experienced the same temperature, humidity, and light cycles as they did as larvae, until they eclosed. We lightly sprayed the pupal containers with water each day. We recorded the length of the pupal stage, eclosion success, and the adult sex for each individual. Adults were euthanized on the day of eclosion in −30°C freezer and stored for two weeks in the pupal containers. Each adult was then pinned and preserved by drying at room temperature.

### 2.4 Photography and image processing

We took high‐resolution macro images of each individual adult moth's wings with the BK Plus System, Bun, Inc., using a Canon® EOS 5Dsr camera with a 100 mm Canon® MP‐E lens. Images were taken in Capture One Pro® (v 10.2.1) using Cam‐Lift Controller (v 1.04), and white‐balanced and cropped in Adobe® Photoshop® (v 19.1.5). For the analysis of seasonal differences in a wild population, we assessed the fore‐ and hindwings and chose either the right‐side or left‐side sets of wings, depending on which ones were better preserved (left wing: *n* = 115, right wing: *n* = 105). Because we found a high correlation in fore‐ and hindwing color patterns (see Supplementary material), we photographed only forewings of the moths from the rearing experiment (left wing: *n* = 114, right wing: *n* = 138). Some of the specimens were damaged or badly angled during pinning (*n* = 72), and some did not eclose properly (*n* = 5), and therefore, we had to discard 77 images (20°C: 36 images, 24°C: 41 images). However, because most of the images were discarded without a biological reason (because of badly pinned specimens), this should not create any systematic biases that could influence our conclusions. The final sample sizes for image analyses were 220 images when analyzing seasonal differences in a wild population (spring season: *n* = 78, summer season: *n* = 142), and 252 images in the rearing experiment (20°C: *n* = 120, 24°C: *n* = 132, 28°C = no larvae survived).

Forewing lengths were measured from base to the apex of the wing using ImageJ (v 1.52a). Fore‐ and hindwing images were then processed with pavo (v 2.0) using the color adjacency package (Maia et al., 2019) in Rstudio, (v 3.4.2, R Core Team, 2019). We generated an index of color proportion, “*pSpot*,” which was the ratio of the orange spot area to the entire wing area, assuming that higher *pSpot* values indicate higher conspicuousness. Although some *A. nigriceps* individuals have additional small orange spots in their wings, most variation in wing coloration results from differences in spot size, rather than different spot arrangements (Figure 1, see Supplementary material for details), and *pSpot* therefore provides a good measure for warning signal expression. Many images had portions of wing area incorrectly designated as either background or wing spots in pavo. These images were subsequently re‐processed in Photoshop® using the “Clone Stamp” tool and then re‐analyzed with pavo.

### 2.5 Statistical analysis

#### 2.5.1 Seasonal differences in a wild population

Differences in warning signal expression between spring (early season Oct–Dec) and summer (late season Feb–April) emergence periods and the wing length of wild specimens from the Macquarie population were analyzed using generalized linear models. The response variables in the models included the proportion of orange in the wing and the wing length (mm), and explanatory variables were an individual's sex and the emergence season. The two emergence seasons were used as a proxy for temperature due to the different environmental temperatures experienced by the larvae during development. Because these seasonal effects might differ between females and males or vary across years (2017–2020), we also ran models that included an interaction between sex and the collection season, or year and the collection season.

#### 2.5.2 Rearing experiment: The effect of temperature

We used a mixed‐effects Cox proportional hazards model to test the effect of temperature on larval survival in the rearing experiment. To investigate possible differences in survival (days before death) among the three populations, explanatory variables in the model included an interaction between the temperature treatment (20/24/28°C, treated as a continuous variable) and population (Macquarie//Wyoming/Mt Tomaree), with family included as a random effect. The larvae that pupated successfully were right‐censored. Differences in eclosion success of pupae in the 20°C and 24°C treatment were compared using a chi‐square test.

The effect of rearing temperature on life‐history traits and warning signal expression was analyzed using generalized linear mixed models with (i) developmental time from larva to pupa (days), (ii) developmental time from pupa to eclosion (days), (iii) pupal weight (g), (iv) adult wing length (mm), or (v) proportion of orange in the forewing as a response variable. To investigate whether individuals from different populations responded to the temperatures differently, explanatory variables in the models included an interaction between the temperature treatment and the population (both categorical variables). The models also included sex as a fixed effect and family as a random effect. In all cases, the significance of the interaction terms was assessed using likelihood‐ratio tests comparing models with and without the interaction term. All analyses were conducted with software R.3.6.1 (R Core team 2019) using lm4 (Bates et al., 2015), lmerTest (Kuznetsova et al., 2017), and coxme (Therneau, 2018) packages.

#### 2.5.3 Rearing experiment: Genetic variance and heritability

We estimated genetic variance and heritability for the wing color trait using two complementary approaches. The first approach consisted of a general linear mixed model (GLMM) in which genetic variance was estimated according to the random animal term (i.e., the “animal model” approach; Lynch & Walsh, 1998, Kruuk, 2004; Wilson et al., 2010). This term represents a pedigree‐wide relationship matrix calculated according to the expected additive genetic relatedness among all individuals in the design (in our case, the relevant coefficients of relatedness are 0.5 for parents–offspring and 0.5 between any two full siblings: Falconer, 1981; Lynch & Walsh, 1998). We, therefore, included all phenotyped individuals in this analysis, including those for all 15 dams and for 11 sires that could be reliably measured. The magnitude of the animal term effect is taken to represent additive genetic variance (*V*_*A*_) and is expressed relative to residual variance (*V*_*R*_) to yield narrow‐sense heritability (*h* ^*2*^) according to the standard equation of *h* ^2^ *= V*_*A*_ */(V*_*A*_ *+ V*_*R*_ *)* (Falconer, 1981). We also included fixed effects of sex, population, and temperature treatment, plus a term coded for generation. The latter variable was included to account for any systematic deviation in the phenotypes of parents (who developed under largely unknown field conditions) from the phenotypes of their laboratory‐bred offspring. We used likelihood‐ratio tests to assess differences in variances between males and females, wherein the overall fit of a model with these variances constrained to equality was compared with a model wherein they were free to vary (Kruuk, 2004, Wilson et al., 2010). The same approach was used to test whether the intersexual genetic correlation varied significantly from 1.0. All such tests involved a constraint to one parameter in the solution and were assessed according to the chi‐squared distribution with one degree of freedom. Animal modeling was conducted using stand‐alone ASReml v4.2 software (Gilmour et al., 2015).

The second approach consisted of regressing parental values upon offspring values. We conducted these because the relationship matrix used to estimate animal model terms (as above) is dominated by full‐sibling relationships. This means that animal modeling estimates could, therefore, be inflated by non‐additive genetic variances (such as dominance and epistasis variance), common environment effects, and maternal effects (Falconer, 1981). The parent–offspring regression is by contrast influenced only by additive genetic variation and, therefore, yields an unbiased estimate of narrow‐sense heritability (*h* ^*2*^; an estimate that excludes maternal, common environment, and non‐additive genetic effects). We regressed mid‐parental values (dam/sire averages) upon offspring means (daughters & sons combined) as well as upon the means for sons and daughters separately. Narrow‐sense heritability is given in all these cases directly by the least‐squares regression slope (Falconer, 1981, Lynch & Walsh, 1998). In calculating mid‐parent values, we excluded cases where sire values were absent, and likewise for offspring values, we excluded cases involving only one sex (ultimate sample sizes are given in the Results). Although relatively lacking in statistical power, these regressions offered a basis for interpreting the additive genetic contribution to our animal modeled heritability estimate.

## 3 RESULTS

### 3.1 Seasonal differences in a wild population

**FIGURE 2:**
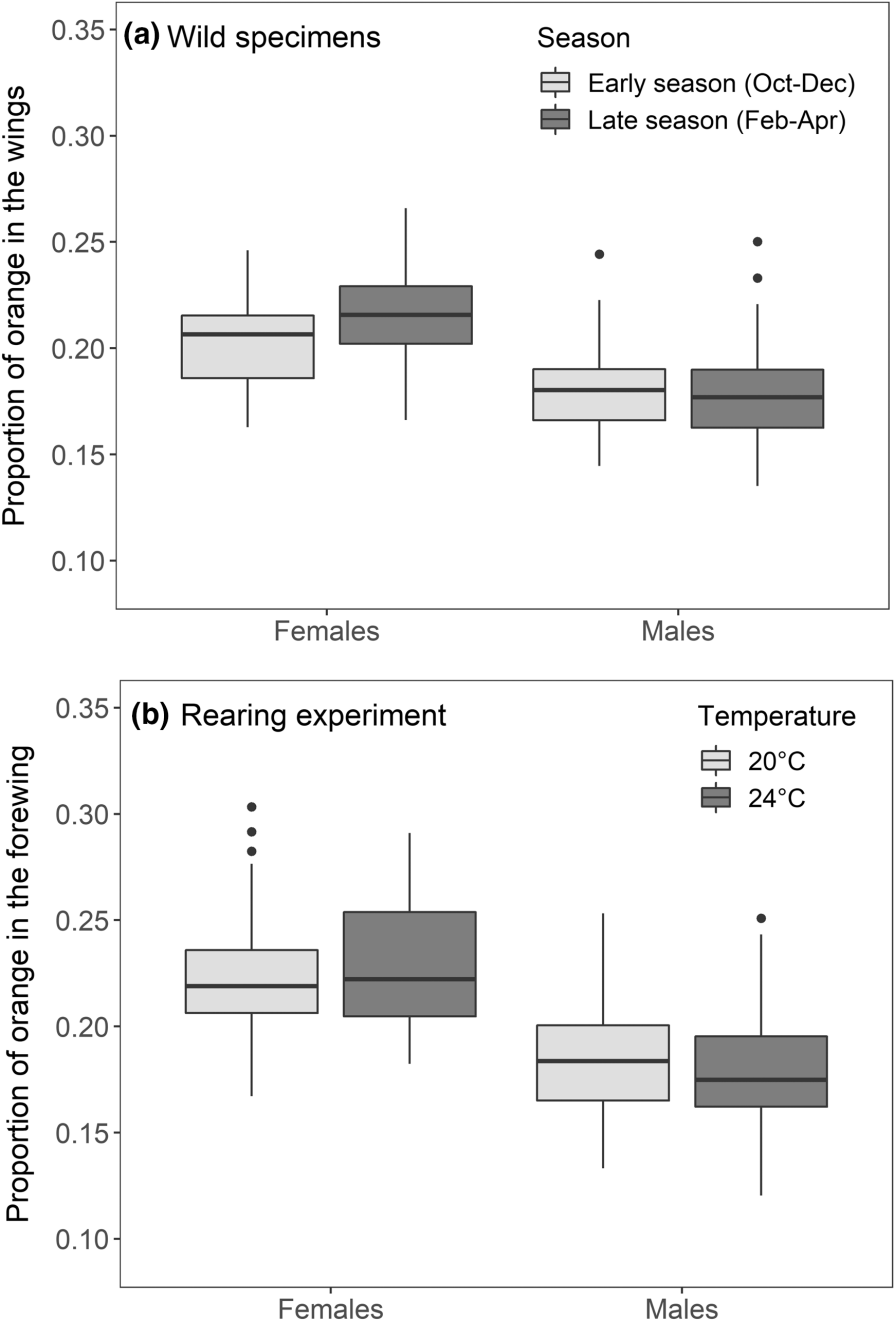
Variation in warning signal expression in *Amata nigriceps* females and males. (a) A proportion of orange in the fore‐ and hindwings of individuals collected from the Macquarie population during the spring (light gray plots, *n* = 78) or summer emergence periods (dark gray plots, *n* = 142) between 2017 and 2020. (b) A proportion of orange in the forewings of individuals reared in 20°C (light gray plots, *n* = 120) or 24°C temperature (dark gray plots, *n* = 132). Box plots indicate the median and 25th and 75th percentiles, the whiskers show the range of values within 1.5 times the interquartile range, and circles represent outliers

The proportion of orange in the combined fore‐ and hindwings varied from 0.14 to 0.27 (mean = 0.19) among individuals collected from the Macquarie population between years 2017 and 2020. However, there was no difference in wing color patterns between individuals collected during the spring and summer seasons (estimate = 0.0004 ± 0.003, *t* = 0.139, *p* = .89; Figure 2a). This result was consistent across years (year × season: estimate = 0.003 ± 0.003, *t* = 0.917, *p* = .36) and in both sexes (sex × season: estimate = 0.013 ± 0.007, *t* = 1.782, *p* = .076), and the interaction terms were not included in the final model. Regardless of the season, females had a higher proportion of orange in the wings compared with males (estimate = 0.033 ± 0.003, *t* = 9.964, *p* < .001; Figure 2a).

Individuals collected during the spring season tended to have longer wings than individuals collected during the summer season; however, this difference was not significant (estimate = 0.292 ± 0.157, *t* = 1.861, *p* = .064). This seasonal pattern was similar in both sexes (sex × season: estimate = 0.180 ± 0.397, *t* = 0.453, *p* = .65) and across years (year × season: estimate = 0.110 ± 0.155, *t* = 0.710, *p* = .48), and these interaction terms were removed from the final model. There were no differences in wing length between females and males (estimate = 0.089 ± 0.178, *t* = 0.499, *p* = .62).

### 3.2 Rearing experiment

#### 3.2.1 Effect of temperature on survival

**FIGURE 3:**
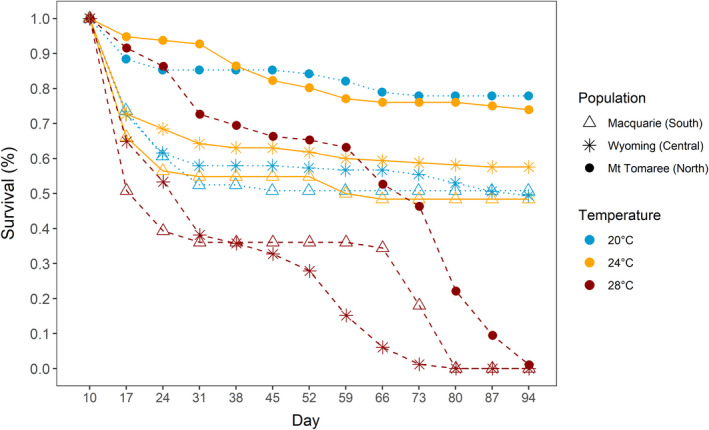
Larval (*n* = 964) survival across days of the rearing experiment. Larvae from three populations (triangles = Macquarie (South), stars = Wyoming (Central), circles = Mt Tomaree (North)) were divided into three different rearing temperatures: 20°C (blue dotted lines), 24°C (orange solid lines) and 28°C (red dashed lines), and their survival was recorded once a week. By day 94 of the experiment, 98% of the larvae had either died or successfully pupated

There was no difference in larval survival between 20°C and 24°C treatments (Figure 3, see Table S2), with 57% survival to the pupal stage at 20°C temperature (*n* = 183 pupae) and 59% at 24°C temperature (*n* = 190 pupae). In contrast, none of the larvae in the 28°C treatment survived to the pupal stage, resulting in significantly lower survival compared with the 20°C and 24°C treatments (effect of temperature: estimate = 0.211 ± 0.015, Z = 14.38, *p* < .001; Figure 3). This effect was similar in all populations (population × temperature treatment: χ2 = 0.951, *df* = 2, *p* = .62), but we found that larvae from Mt Tomaree population had overall a higher survival compared with the larvae from the Macquarie (estimate = 1.440 ± 0.606, Z = 2.37, *p* = .018) or the Wyoming populations (estimate = 1.179 ± 0.472, Z = 2.50, *p* = .012; Figure 3).

There was no difference in eclosion success between the 20 and 24°C treatments (Chi‐square test: χ^2^ = 0.198, *df* = 1, *p* = .66). In total, 156 pupae (91%) eclosed successfully in the 20°C treatment (Macquarie: *N* = 24, Wyoming: *N* = 69, Mt Tomaree: *N* = 63) and 173 pupae (93%) in the 24°C treatment (Macquarie: *N* = 24, Wyoming: *N* = 87, Mt Tomaree: *N* = 62).

#### 3.2.2 Effect of temperature on life‐history traits

**FIGURE 4:**
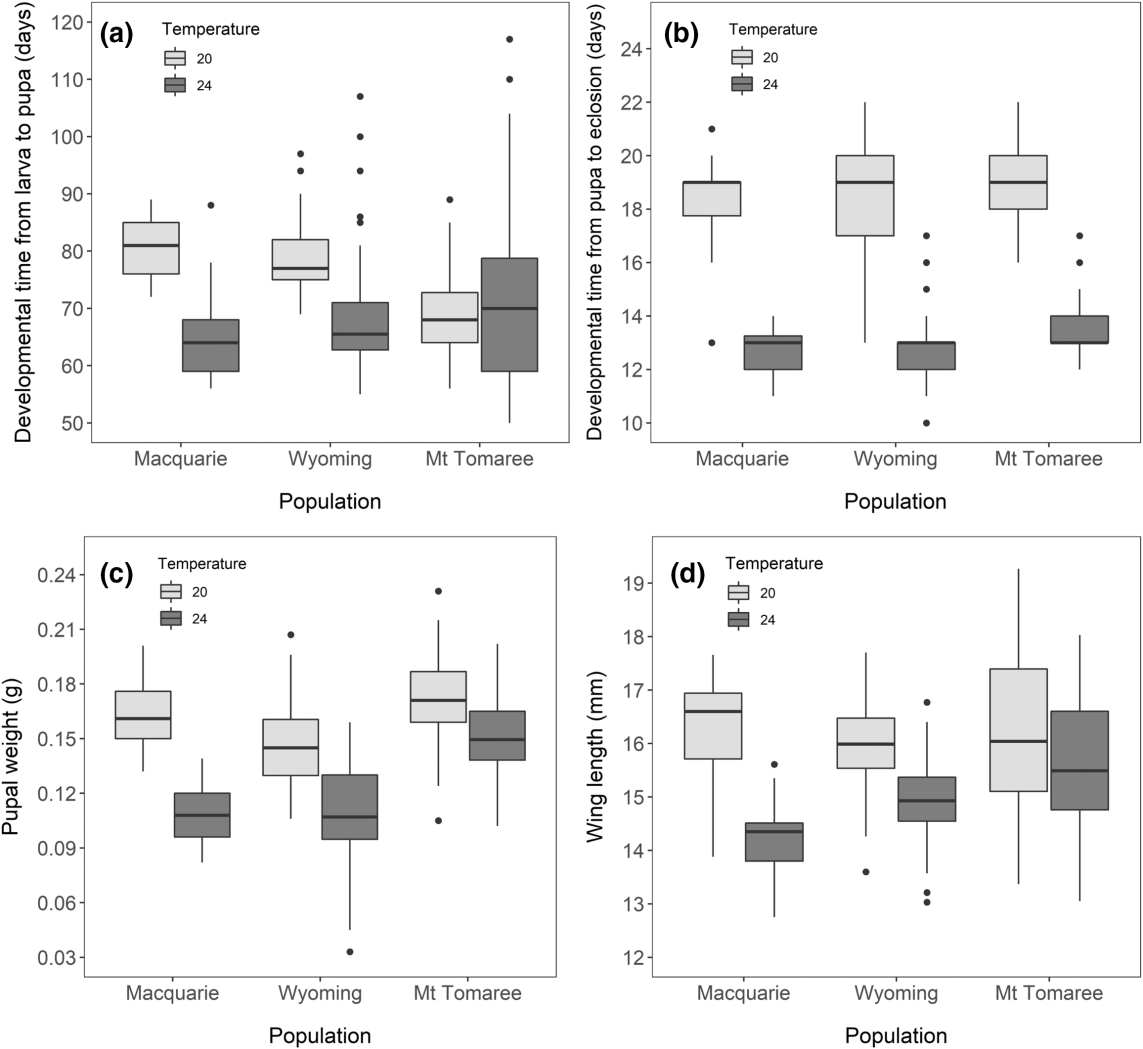
Effect of temperature on life‐history traits. The graphs show the effect of temperature on (a) developmental time from larva to pupa (*n* = 358), (b) developmental time from pupa to eclosion (*n* = 328), (c) pupal weight (*n* = 358), and (d) adult wing length (*n* = 252). Individuals were collected from three different populations and reared in different temperatures (light gray plots = 20°C treatment, dark gray plots = 24°C treatment). Box plots indicate the median and 25th and 75th percentiles, the whiskers show the range of values within 1.5 times the interquartile range, and circles represent outliers. One outlier (individual that eclosed in 7 days) was excluded from (b) as this was likely to be a recording error

The effect of temperature on developmental time from larva to pupa differed among the three populations (population × temperature treatment: χ^2^ = 54.47, *df* = 2, *p* < .001 Figure 4a). Larvae from the Macquarie and Wyoming populations took longer to pupate in the 20°C treatment compared with the 24°C treatment (Macquarie: estimate = 16.184 ± 2.344, *t* = 6.905, *p* < .001; Wyoming: estimate = 9.506 ± 1.301, *t* = 7.307, *p* < .001). However, this difference was not found in larvae from the Mt Tomaree population (estimate = −2.109 ± 1.429, *t* = −1.476, *p* = .14; Figure 4a). Temperature also influenced the time from pupa to eclosion, with longer pupation times in the 20°C than in the 24°C treatment (estimate = 5.727 ± 0.127, *t* = 45.212, *p* < .001; Figure 4b). Regardless of the temperature treatment (population × temperature treatment: χ^2^ = 0.035, *df* = 2, *p* = .98), individuals from the Mt Tomaree population had longer pupation times compared with two other populations (compared with Macquarie: estimate = 0.983 ± 0.337, *t* = 2.919, *p* = .014; compared with Wyoming: estimate = 0.812 ± 0.230, *t* = 3.523, *p* = .005), although these differences were biologically small (Figure 4b). In addition, pupation time was longer in males than in females (estimate = 1.393 ± 0.127, *t* = 10.966, *p* < .001).

Rearing temperature also influenced the size of the moths. Pupae in the 20°C treatment were heavier than pupae in the 24°C treatment in all three populations (all *p* < .001; Figure 4c). Similarly, moths reared at 20°C had longer wings compared with moths in the 24°C treatment (all *p* < .001; Figure 4d). However, the size of the effect varied among populations in both pupal weight (population × temperature treatment: χ^2^ = 36.88, *df* = 2, *p* < .001) and in wing length (population × temperature treatment: χ^2^ = 17.525, *df* = 2, *p* < .001), with the smallest effect of temperature found in the Mt Tomaree population, and the largest effect in the Macquarie population (Figure 4, see Supplementary material for full model outputs). In addition, pupal weights differed among the populations, with pupae from Mt Tomaree being generally heavier compared with the other two populations (Figure 4c, see Supplementary material). We also found that females were heavier (estimate = 0.027 ± 0.002, *t* = 16.386, *p* < .001) and had shorter wings compared with males (estimate = −1.364 ± 0.097, *t* = 13.990, *p* < .001).

#### 3.2.3 Effect of temperature on warning signal expression

The proportion of orange in the forewing varied from 0.12 to 0.30 among individuals (mean = 0.20), but this was not influenced by the temperature treatment (estimate = 0.001 ± 0.002, *t* = 0.485, *p* = .63; Figure 2b). The effect was similar in all populations (population × temperature treatment: χ^2^ = 3.637, *df* = 2, *p* = .16), so the interaction term was removed from the final model. However, regardless of the temperature, moths from the Mt Tomaree population had a higher proportion of orange in their forewings compared with the Macquarie (estimate = 0.033 ± 0.014, *t* = 2.322, *p* = .038) or the Wyoming populations (estimate = 0.024 ± 0.010, *t* = 2.416, *p* = .032). In addition, females had a higher orange/black ratio compared with males (estimate = 0.043 ± 0.002, *t* = 17.381, *p* < .001; Figure 2b).

#### 3.2.4 Heritability of the warning signal

**FIGURE 5:**
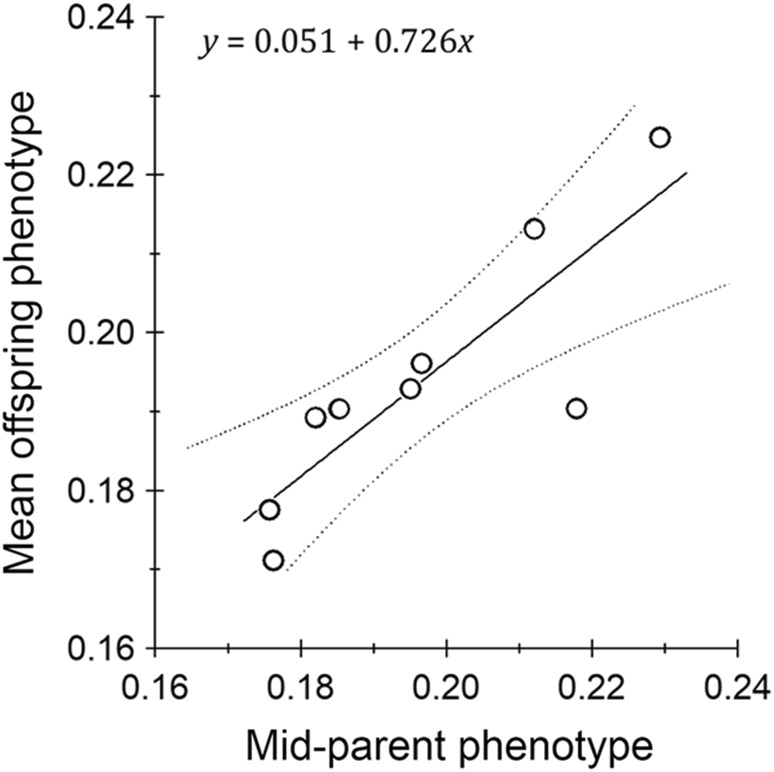
Mid‐parent–offspring regression performed to estimate narrow‐sense heritability (*h^2^*) for warning signal expression in *Amata nigriceps* (i.e., a proportion of orange in the wings). The fitted line is a least‐squares regression as described by the equation indicated in the panel and is shown with 95% regression bands

Animal modeling for warning signal expression indicated no differences between the sexes for genetic variance (*V*_*G* (females)_ = 5.12 ± 1.95; *V*_*G* (males)_ = 3.35 ± 1.35; *G*_*1*_ = 0.332, *p* = .564) or residual variance (*V*_*R* (females)_ = 0.45 ± 1.03; *V*_*R* (males)_ = 2.20 ± 0.81; *G*_*1*_ = 2.342, *p* = .126), and an intersexual genetic correlation was not significantly different from 1.0 (*R*_*G*_ = 0.890 ± 0.130; *G*_*1*_ = 1.102, *p* = .294). The subsequently sex‐pooled estimates for genetic variance (*V*_*G*_ = 3.83 ± 1.26) and residual variance (*V*_*R*_ = 1.66 ± 0.65) both significantly exceeded zero (*G*_*1*_ > 19.0, *p* < .001). These values yielded an overall heritability estimate of *H* ^2^ = 0.698 ± 0.148, which agreed closely with the estimate gained from mid‐parent–offspring regression (*h* ^2^ = 0.726 ± 0.170; *n* = 9; Figure 5), suggesting that the warning signal is highly heritable. The similarity of these two estimates implies the absence of significant common environment and non‐additive genetic effects. Further, separate regressions of mid‐parents upon sons (*h* ^2^ = 0.705 ± 0.215; *n* = 10) and daughters (*h* ^2^ = 0.709 ± 0.160; *n* = 10) yielded almost identical estimates, which agrees with the animal model finding that genetic and residual variances were equivalent among the sexes. Full regression results are given as Supplementary Online information.

## 4 DISCUSSION

Understanding what maintains the observed diversity in warning signals in aposematic species requires us to examine both environmental and genetic components of variation. Here, we investigated this in the aposematic moth, *Amata nigriceps*. We found that the warning signal size in the moths was not affected by the different environmental temperatures experienced during development in the wild or by the temperature treatment in a full‐sibling laboratory experiment. Instead, we found that 70% of the phenotypic variation in the proportion of orange in the warning signal of *A. nigriceps* arose due to genetic differences among individuals. This is similar to moderate‐to‐high heritability reported for the warning signals of other aposematic species (e.g., Burdfield‐Steel & Kemp, 2021; Lindstedt et al., 2009) and provides insight to the basis of the continuous variation in warning coloration in *A. nigriceps* populations.

We found a high and significant genetic component to the variation in proportion of orange in the moth warning signals. In some aposematic species, warning signals are negatively genetically correlated across the sexes (Burdfield‐Steel & Kemp, 2021), but we did not find evidence that the sexes expressed different levels of genetic variance, and the intrasexual genetic correlation was estimated to not deviate from one. The fact that heritability as estimated from animal modeling (where most of the relationship matrix is dominated by full siblings) was closely replicated by parent–offspring regression implicates additive genes as the primary source of variation (Falconer, 1981). This conclusion is further supported by the lack of any sex difference in genetic variation and heritability—particularly as estimated by parent–offspring regression—which suggests the absence of maternal effects. Overall, heritability was notably high (*H* ^2^ ~ *h* ^2^ ~ 0.7), which is commensurate with heritability estimates gained for wing color characters in other lepidopteran species (e.g., Kemp & Rutowski, 2007; Kingsolver & Wiernasz, 1991). The large basis of quantitative genetic variation in *A. nigriceps* is somewhat intriguing given that aposematic color patterns are thought to experience stabilizing selection (Endler, 1988; Lindström et al., 2001; Mallet & Joron, 1999; Rowland et al., 2010). This finding recasts the question of what maintains phenotypic variation in this species to what maintains the extensive genetic variation which underpins it.

Coloration often has an important function in thermoregulation, and thermal benefits might maintain variation in warning signal expression as demonstrated in the aposematic wood tiger moth, *Arctia plantaginis* (Hegna et al., 2013; Lindstedt et al., 2009). However, we found no evidence of temperature‐induced plasticity in the warning signals of wild‐caught or laboratory‐reared *A. nigriceps*, which differs from several experiments that have demonstrated that low rearing temperatures increase melanization in other lepidopteran species (e.g., Davis et al., 2005; Karl et al., 2009; Lewis, 1985; Rosa & Saastamoinen, 2020, but see Forsman, 2011 for contrasting results in grasshoppers). It is possible that seasonal differences of eight degrees in our collection site, or temperature differences of four degrees in our rearing experiment, were too small to induce plastic responses in *A. nigriceps*. To better understand temperature‐driven selection pressures and the thermal benefits of melanization in the species, future studies should include a broader range of temperatures, including the most extreme temperature regions of Australia, as well as directly measure the potential fitness benefits of melanin in colder temperatures (Clusella‐Trullas et al., 2007). Similarly, further work is needed to understand potential fitness benefits of coloration in hot temperatures as darker coloration might also play an important role in UV protection, desiccation resistance and immunity (Bastide et al., 2014; Friman et al., 2009; Parkash et al., 2008).

Consistent with previous work (Binns et al. in review), we found that females had a higher proportion of orange in the wings compared with males. More conspicuous warning signals are expected to increase the speed and strength of avoidance learning and result in fewer recognition errors in predators (Roper & Redston, 1987; Speed, 2000). Larger orange spots might, therefore, be a stronger warning signal to predators, which could be more beneficial for females that are heavier and, therefore, provide a greater nutritional reward. Predator community composition might also explain population‐level differences in warning coloration (Endler & Mappes, 2004; Nokelainen et al., 2014), with a previous study finding a correlation between *A. nigriceps* warning signals and invertivore diversity in the specific moth collection site (Binns et al. in review). We found that individuals from Mt Tomaree population had less melanized (more orange) wings compared with the other two populations. Besides possible differences in a predator community, this could be explained by abiotic differences (e.g., higher rainfall, Parkash et al., 2008), but more controlled experiments with a higher number of study sites are needed to better understand these environmental effects.

Although we did not find evidence of temperature influencing warning signal expression, it affected larval survival, growth, and development in our rearing experiment, with none of the larvae at the highest 28°C temperature surviving until pupation. Temperatures above 30°C have been shown to reduce survival in other Australian lepidopterans (Jones et al., 1987), but our result is surprising, given that summer temperatures in our moth collection sites in Sydney commonly exceed 28°C (http://www.bom.gov.au/climate/data/). The highest temperature treatment was, therefore, in the range that occurs in *A. nigriceps* broad habitat, but it is possible that in the wild larvae move to cooler and more moist microhabitats, than the conditions available in our experiment. We also found that *A. nigriceps* had longer developmental times and larger body sizes in the lower temperature treatment. The same pattern has been observed in many ectotherms, and the tendency of individuals to grow more slowly and mature at larger body size is termed the “temperature size rule” (TSR, Atkinson, 1994; Zuo et al., 2012). Interestingly, this effect was smaller in individuals from Mt Tomaree population, and larvae from Mt Tomaree also had higher survival compared with the other two populations. In addition, adult moths from Mt Tomaree population differed from the other two populations phenotypically, having larger body sizes and a higher proportion of orange in the wings. Whether this represents a GxE interaction or local adaptation warrants further study, which will also clarify whether *A. nigriceps* are part of a group that contains several subspecies (Marriott, 2014).

## 5 CONCLUSION

Our study highlights the importance of considering both genetic differences and plastic responses to the environment if we want to understand what maintains intraspecific variation in aposematic species. We showed that *A. nigriceps* warning signals are highly heritable, but further research is needed to understand how different selection pressures on signaling and non‐signaling functions of the coloration contribute to the genetic variation. This includes testing how the observed variation in *A. nigriceps* warning signals influences predators' attack decisions and learning (Rowe et al., 2004; Rowland et al., 2010), whether more melanic warning signals act as defense against pathogens (Friman et al., 2009; Zhang et al., 2012), and how these might trade off with thermoregulation or desiccation (Hegna et al., 2013; Lindstedt et al., 2009; Parkash et al., 2008). Identifying the chemical defense of *Amata* species and quantifying the costs of these defenses and signaling (e.g., warning signal honesty, Summers et al., 2015) will be an important addition to this discussion. Finally, the inheritance of warning signals may correlate genetically with other morphological and life‐history traits (Evans, 2010), and to understand variation in warning signal expression, we therefore need to study the patterns of genetic variation and covariation underlying those traits.

## AUTHOR CONTRIBUTIONS

**Georgina Erika Binns:** Conceptualization (equal); data curation (equal); investigation (equal); methodology (equal); validation (equal); writing – original draft (equal); writing – review and editing (equal). **Liisa Hämäläinen:** Conceptualization (equal); data curation (equal); formal analysis (equal); investigation (equal); methodology (equal); software (equal); validation (equal); visualization (equal); writing – original draft (equal); writing – review and editing (equal). **Darrell Kemp:** Formal analysis (equal); software (equal); visualization (equal); writing – original draft (equal); writing – review and editing (equal). **Hannah M. Rowland:** Funding acquisition (equal); validation (supporting); writing – review and editing (supporting). **Kate Umbers:** Funding acquisition (equal); writing – review and editing (supporting). **Marie Herberstein:** Conceptualization (equal); funding acquisition (equal); project administration (equal); supervision (equal); validation (equal); writing – review and editing (equal).

## CONFLICT OF INTEREST

The authors have no conflicts of interest to declare.

## DIVERSITY STATEMENT

We strongly support Equity, Diversity, and Inclusion in Science (Rößler et al., 2020). Researchers included in this study are in different stages of their careers, from PhD student through to Early, Mid, and Senior researchers. Our gender balance is biased toward women, and at least two authors identify as part of the LGBTQIA+ community. Our authors come from different backgrounds such as Austria, Australia, Finland, and the United Kingdom.

## Supporting information


supporting Information

## Data Availability

https://doi.org/10.5061/dryad.prr4xgxpn
