# Supplementary material for: Additive genetic variation, but not temperature, influences warning signal expression in Amata nigriceps moths (Lepidoptera: Arctiinae)
Source: Ecol Evol. 2022 Jul 17;12(7):e9111. doi: 10.1002/ece3.9111 (PMC9288930; doi:10.1002/ece3.9111)
Supplement: Supplementary file 1 — supporting Information [file ECE3-12-e9111-s001.docx]

**Supplementary material for**

**Additive genetic variation, but not temperature, influences warning signal expression in *Amata nigriceps* moths (Lepidoptera: Arctiinae)**

Authors: Georgina E. Binns^1*^, Liisa Hämäläinen^1*^, Darrell J. Kemp^1^, Hannah M. Rowland^2^, Kate D. L. Umbers^3,4^, and Marie E. Herberstein^1^

^1^School of Natural Sciences, 14 Eastern Road, Macquarie University, North Ryde, 2109 NSW Australia

^2^ Max Planck Institute for Chemical Ecology, Hans Knöll Straße 8, 07749 Jena, Germany

^3^ School of Science, Western Sydney University, Penrith, 2751 NSW, Australia

^4^ Hawkesbury Institute for the Environment, Western Sydney University, Penrith, 2751 NSW, Australia

*Joint first authors

Corresponding author: Georgina E. Binns, School of Natural Sciences, Macquarie University, 14 Eastern Road, North Ryde 2109, NSW, Australia. E-mail: georgina.binns@mq.edu.au

**Contents**

**Supplementary methods2**

1. Correlation between fore- and hindwing colouration 2
2. The number of orange wing spots3
3. Moth collection sites4
4. Family representation in the rearing experiment4

**Supplementary Results: Full model outputs6**

1. Seasonal differences in a wild population 6
2. Rearing experiment: survival, life-history traits and warning signal expression 6
3. Rearing experiment: genetic basis of warning signal expression 9

a) GLMM approach (i.e., the ‘animal model’ approach) 9

b) Parent – offspring regression approach 11

**SUPPLEMENTARY METHODS**

**1. Correlation between fore- and hindwing colouration**

To investigate whether *pSpot* values (proportion of orange) of both forewings and hindwings were comparable with the *pSpot* values of forewings only, we randomly chose 60 images of wild specimens collected from the Macquarie population (30 females and 30 males). We cropped hindwings out of these images, and then re-processed them with pavo (following the methods described in the main text). The *pSpot* values of forewings were then compared to the values of both wings. We found a strong positive correlation between these measures (Pearson correlation: r = 0.935, t = 20.085, df = 58, p < 0.001; Fig. S1), and the range of *pSpot* values was very similar in both cases (both wings: min = 0.149, median = 0.187, max = 0.250; forewing only: min = 0.146, median = 0.185, max = 0.253). Our analysis therefore suggested that wing pattern measures of the set of both wings and the forewings only were comparable, and we used only forewings when analysing the images of individuals from the rearing experiment.

**
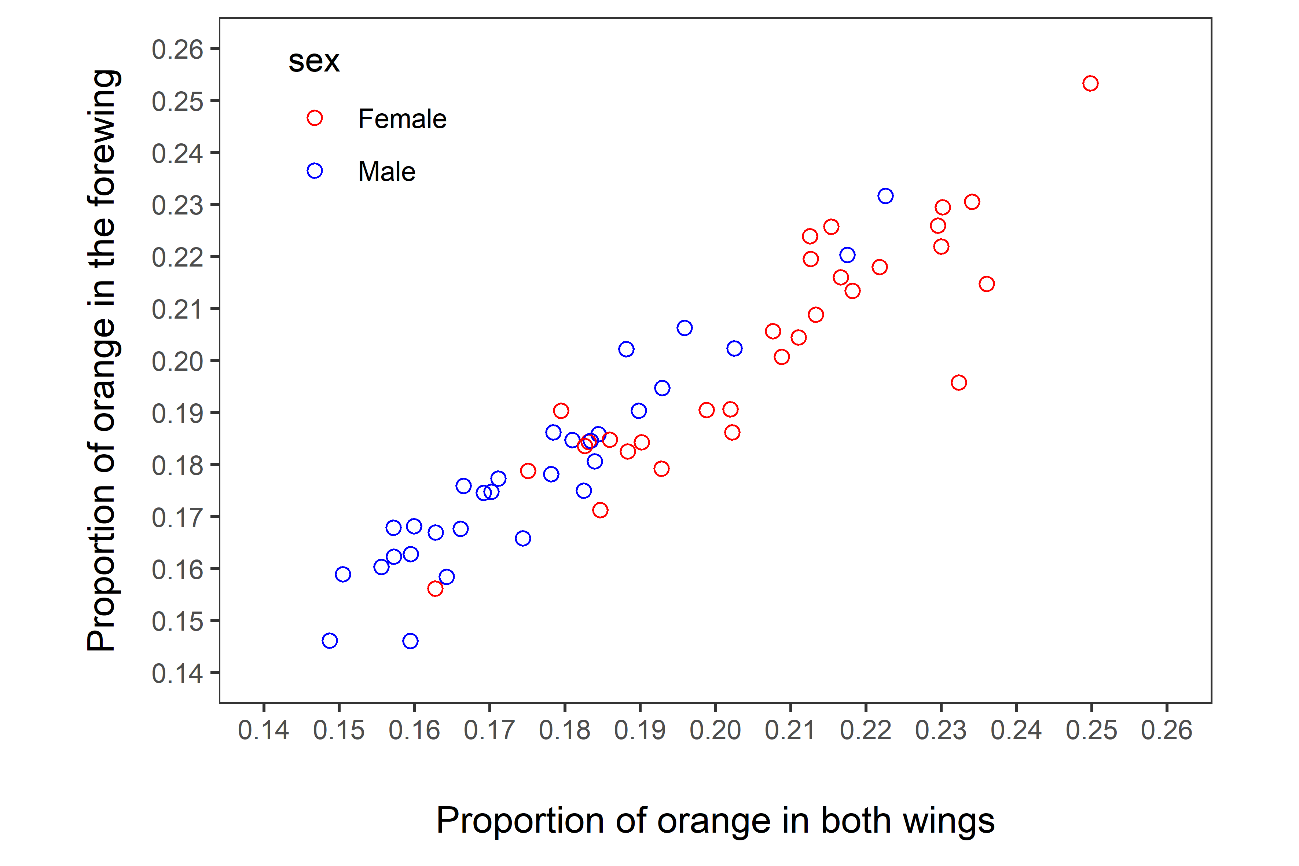
**

**Figure S1.** Correlation between the proportion of orange in the forewings (y axis) and in both fore- and hindwings (x axis) of *A. nigriceps* (n = 60, red dots = females, blue dots = males).

**2. The number of orange wing spots**

We did not quantify the number of orange wing spots in the current study, but we have previously investigated this in 164 *Amata nigriceps* specimens that were collected from New South Wales between years 2017 and 2019 (22 females, 142 males). The moths were photographed and the proportion of orange in the wings was quantified following the methods described in the main text. In addition, we calculated the number of orange spots in the fore- and hindwings. This varied from 9 to 14 spots among individuals, with the majority of the moths (88%) having 10-12 orange spots (Fig. S2). The spatial arrangement of the larger spots was very consistent among individuals (see Fig. 1 in the main text), but some specimens had one or two small additional spots, or missed one spot. There was a positive correlation between the number of orange spots and the proportion of orange in wing (Pearson correlation: r = 0.565, t = 8.706, df = 162, p < 0.001; Fig. S2), but the number of spots did not explain all observed variation in the wing colouration. For example, the proportion of orange varied from 0.13 to 0.29 among individuals that all had 11 wing spots (Fig. S2), indicating that the variation resulted from the size of the spots, rather than their number. These results suggest that the proportion of orange in the wing is a good estimate for warning signal expression, and we therefore used this measure in our study.

**
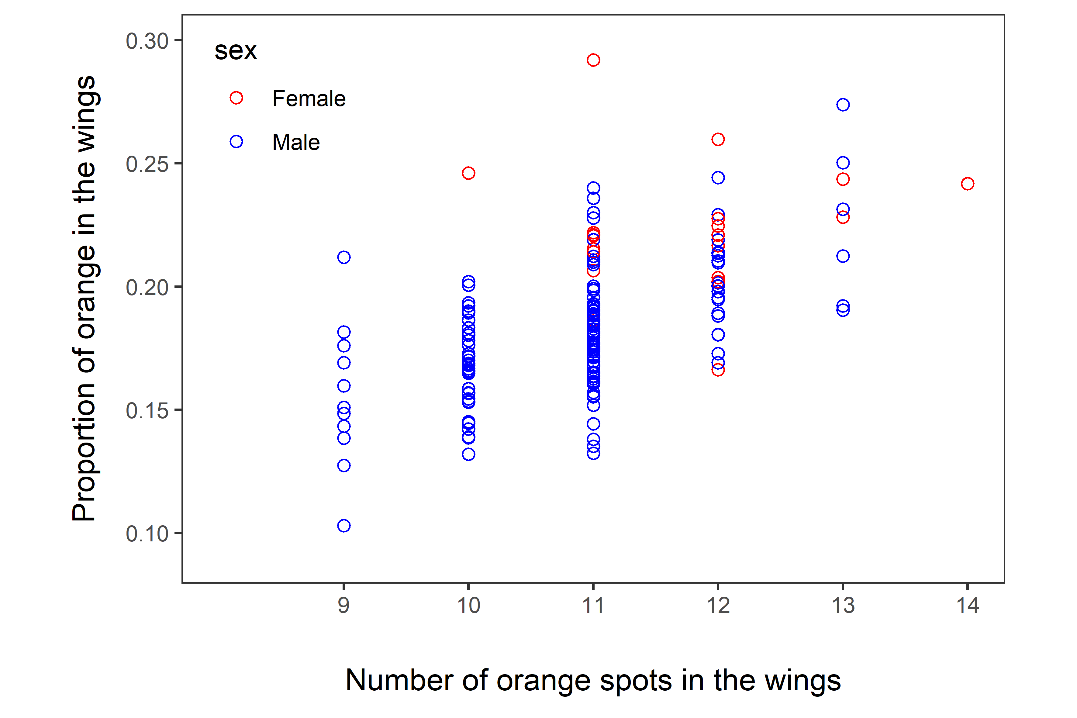
**

**Figure S2.** Correlation between the proportion of orange in the wings and the number of orange wing spots in *A. nigriceps* collected from New South Wales between years 2017 and 2019 (n = 164, red dots = females, blue dots = males). The graph shows a high variation in the proportion of orange in the wings even when the individuals have the same number of orange spots.

**3. Moth collection sites**

We collected mating pairs for the rearing experiment from three locations in New South Wales. Table S1 shows the climate data from these collection sites.

**Table S1.** Differences in local temperature and rainfall between three collection sites, Macquarie Park, Wyoming, and Mt Tomaree, NSW. Mean figures taken from two months (August, September) prior to adult flight season, when *A. nigriceps* is assumed to be in larval stage. Data downloaded from Australian Bureau of Meteorology ([http://www.bom.gov.au/climate/data/](about:blank)) taken from nearest weather stations with 30+ years of climate data (Terrey Hills, Mangrove Mountain and Nelson Bay, respectively).

| Location | Latitude/Longitude | Mean Max Temp during Larvae Season | Mean Min Temp during Larvae Season | Mean Rainfall during Larvae Season |
| --- | --- | --- | --- | --- |
| Macquarie Park | 33°46’25.77” S 151°06’45.54” E | 19.25°C | 9.7°C | 58.05mm |
| Wyoming | 33°24’23.39” S 151°21’37.60” E | 19°C | 6.4°C | 63.2mm |
| Mt Tomaree | 32°43’05.48” S 152°10’58.58” E | 20.2°C | 11°C | 95.3mm |

**4. Family representation in the rearing experiment**

When assigning larvae to the rearing experiment, we took all larvae from the first two egg batches that hatched (MT-02: 120 larvae and MQU-03: 93 larvae, see Table S2). After that we randomly selected 60 individuals from each batch (20 for each temperature). However, in seven batches (2 from Macquarie, 5 from Wyoming and 1 from Mt Tomaree), there were less than 60 larvae alive after 10 days, in which case we took all individuals (ranging from 19 to 53 larvae, see Table S2). In three cases (out of 20 families), all larvae in the egg batch were dead after 10 days (all from Wyoming), which resulted a final sample size of 17 families.

**Table S2. Family representation in the rearing experiment.**

| **Family *** | **No. of larvae hatched** | **No. of larvae alive after 10 days** | **No. of larvae in the experiment**    **20°C 24°C 28°C** | | |
| --- | --- | --- | --- | --- | --- |
|  |  |  |  |  |  |
| MT-01  MT-02  MT-03  MT-04  MQ-03  MQ-04  MQ-05  WY-01  WY-02  WY-03  WY-04  WY-06  WY-07  WY-08  WY-09  WY-10  WY-11 | NA  NA  NA  NA  120  110  89  83  72  58  37  72  61  67  68  82  72 | 46  120  NA  NA  93  45  46  83  42  53  19  72  61  20  68  82  72 | 15  40  20  20  31  15  15  20  14  17  7  20  20  6  20  20  20 | 16  40  20  20  31  15  16  20  14  18  6  20  20  7  20  20  20 | 15  40  20  20  31  15  15  20  14  18  6  20  20  7  20  20  20 |

***** MT = Mt Tomaree population, MQ = Macquarie population, WY = Wyoming population

NA = the total number of larvae was not recorded

**SUPPLEMENTARY RESULTS: FULL MODEL OUTPUTS**

**1. Seasonal differences in a wild population**

**Table S3.** Best-fit generalized linear model explaining the proportion of orange in the fore- and hindwings of *Amata nigriceps* collected from Macquarie Park, Sydney (n = 220). Explanatory variables in the best-fit model included the collection season (spring Oct-Dec/summer Feb-Apr) and an individual’s sex. Intercept gives the estimate for females collected during spring (Oct-Dec). Residual degrees of freedom = 217.

|  |  |  |  |  |  |  |  |  |
| --- | --- | --- | --- | --- | --- | --- | --- | --- |
| **Terms in the model** |  |  | **Estimate** | **SE** |  | **t** |  | **P** |
| Intercept |  |  | 0.211 | 0.003 |  | 71.759 |  | < 0.001 |
| Season (summer Feb-Apr) |  |  | -0.0004 | 0.003 |  | - 0.139 |  | 0.89 |
| Sex (male) | | | -0.033 | 0.003 |  | -9.964 |  | < 0.001 |

**Table S4.** Best-fit generalized linear model explaining wing length (mm) of *Amata nigriceps* collected from Macquarie Park, Sydney (n = 220). Explanatory variables in the best-fit model included the collection season (spring Oct-Dec/summer Feb-Apr) and an individual’s sex. Intercept gives the estimate for females collected during spring (Oct-Dec). Residual degrees of freedom = 217.

|  |  |  |  |  |  |  |  |  |
| --- | --- | --- | --- | --- | --- | --- | --- | --- |
| **Terms in the model** |  |  | **Estimate** | **SE** |  | **t** |  | **P** |
| Intercept |  |  | 15.412 | 0.160 |  | 96.357 |  | < 0.001 |
| Season (summer Feb-Apr) |  |  | -0.292 | 0.157 |  | -1.861 |  | 0.064 |
| Sex (male) | | | 0.089 | 0.178 |  | 0.499 |  | 0.619 |

**2. Rearing experiment: survival, life-history traits and warning signal expression**

**Table S5**. Best-fit mixed effects Cox proportional hazards model explaining larval survival in the *Amata nigriceps* rearing experiment (n = 964). Time before death (days) was explained by temperature treatment (20/24/28°C, continuous variable) and population (Macquarie/Wyoming/Mt Tomaree), and family was included as a random effect (variance = 0.599).

|  |  |  |  |  |  |  |  |  |
| --- | --- | --- | --- | --- | --- | --- | --- | --- |
| **Terms in the model** |  |  | **Estimate** | **SE** |  | **Z** |  | **P** |
| Temperature |  |  | 0.211 | 0.015 |  | 14.38 |  | < 0.001 |
| Population (Mt Tomaree) |  |  | -1.440 | 0.606 |  | -2.37 |  | 0.018 |
| Population (Wyoming) | | | -0.260 | 0.521 |  | -0.50 |  | 0.620 |

**Table S6**. Best-fit generalized linear mixed model explaining the developmental time from larva to pupa (days) in the *Amata nigriceps* rearing experiment (n = 329). Explanatory variables in the best-fit model included the interaction between rearing temperature (24/28°C) and population (Macquarie/Wyoming/Mt Tomaree), and sex as a fixed effect and family as a random effect (variance = 28.98). Intercept gives the estimate for females from Macquarie population, reared at 20°C. Because we did not know the sex of the pupae that did not eclose (20°C: n = 15, 24°C: n = 14), these individuals were not included in the final analysis. P-values and degrees of freedom were estimated using Satterthwaite’s approximation (*lmerTest* function in R).

| **Terms in the model** | **Estimate** | **SE** | **df** | **t** | **P** |  |
| --- | --- | --- | --- | --- | --- | --- |
| Intercept | 82.023 | 4.234 | 13.251 | 19.372 | < 0.001 | |
| Temperature (24°C) | -16.184 | 2.344 | 314.310 | -6.905 | < 0.001 | |
| Population (Mt Tomaree) | -10.699 | 5.105 | 12.475 | -2.096 | 0.057 | |
| Population (Wyoming) | -2.086 | 4.699 | 13.319 | -0.444 | 0.664 | |
| Sex (male) | -1.419 | 0.894 | 313.862 | -1.587 | 0.114 | |
| Temperature (24°C) * Population (Mt Tomaree) | 18.292 | 2.745 | 313.399 | 6.664 | < 0.001 | |
| Temperature (24°C) * Population (Wyoming) | 6.677 | 2.680 | 313.986 | 2.491 | 0.013 | |

**Table S7.** Best-fit generalized linear mixed model explaining the developmental time from pupa to eclosure (days) in the *Amata nigriceps* rearing experiment (n = 329). Explanatory variables in the best-fit model included rearing temperature (24/28°C), population (Macquarie/Wyoming/Mt Tomaree) and sex, and family as a random effect (variance = 0.086). Intercept gives the estimate for females from Macquarie population that were reared at 20°C. P-values and degrees of freedom were estimated using Satterthwaite’s approximation (*lmerTest* function in R).

| **Terms in the model** | **Estimate** | **SE** | **df** | **t** | **P** |
| --- | --- | --- | --- | --- | --- |
| Intercept | 17.572 | 0.298 | 14.356 | 58.893 | < 0.001 |
| Temperature (24°C) | -5.727 | 0.127 | 319.942 | -45.212 | < 0.001 |
| Population (Mt Tomaree) | 0.983 | 0.337 | 10.761 | 2.919 | 0.014 |
| Population (Wyoming) | 0.171 | 0.316 | 11.710 | 0.542 | 0.598 |
| Sex (male) | 1.393 | 0.127 | 319.282 | 10.966 | < 0.001 |

**Table S8.** Best-fit generalized linear mixed model explaining pupal weight (g) in the *Amata nigriceps* rearing experiment (n = 329). Explanatory variables in the best-fit model included the interaction between temperature (24/28°C) and population (Macquarie/Wyoming/Mt Tomaree), and sex as a fixed effect and family as a random effect (variance = 0.0000094). Intercept gives the estimate for females from Macquarie population that were reared at 20°C. Because we did not know the sex of the pupae that did not eclose (20°C: n = 15, 24°C: n = 14), these individuals were not included in the final analysis. P-values and degrees of freedom were estimated using Satterthwaite’s approximation (*lmerTest* function in R).

| **Terms in the model** | **Estimate** | **SE** | **df** | **t** | **P** | |  |
| --- | --- | --- | --- | --- | --- | --- | --- |
| Intercept | 0.180 | 0.004 | 15.878 | 45.708 | | < 0.001 | |
| Temperature (24°C) | -0.050 | 0.004 | 321.105 | -11.781 | | < 0.001 | |
| Population (Mt Tomaree) | 0.007 | 0.005 | 12.748 | 1.631 | | 0.127 | |
| Population (Wyoming) | -0.019 | 0.004 | 15.340 | -4.485 | | < 0.001 | |
| Sex (male) | -0.027 | 0.002 | 314.734 | -16.386 | | < 0.001 | |
| Temperature (24°C) * Population (Mt Tomaree) | 0.030 | 0.005 | 319.577 | 6.037 | | < 0.001 | |
| Temperature (24°C) * Population (Wyoming) | 0.017 | 0.005 | 320.684 | 3.528 | | < 0.001 | |

**Table S9.** Best-fit generalized linear mixed model explaining adult wing length (mm) in the *Amata nigriceps* rearing experiment (n = 252). Explanatory variables in the best-fit model included the interaction between temperature (24/28°C) and population (Macquarie/Wyoming/Mt Tomaree), and sex as a fixed effect and family as a random effect (variance = 0.115). Intercept gives the estimate for females from Macquarie population that were reared at 20°C. P-values and degrees of freedom were estimated using Satterthwaite’s approximation (*lmerTest* function in R).

| **Terms in the model** | **Estimate** | **SE** | **df** | **t** | **P** | |  |
| --- | --- | --- | --- | --- | --- | --- | --- |
| Intercept | 15.514 | 0.317 | 14.971 | 48.905 | | < 0.001 | |
| Temperature (24°C) | -1.916 | 0.263 | 242.530 | -7.280 | | < 0.001 | |
| Population (Mt Tomaree) | 0.027 | 0.375 | 12.977 | 0.072 | | 0.944 | |
| Population (Wyoming) | -0.208 | 0.349 | 14.320 | -0.597 | | 0.560 | |
| Sex (male) | 1.364 | 0.097 | 238.147 | 13.990 | | < 0.001 | |
| Temperature (24°C) * Population (Mt Tomaree) | 1.271 | 0.311 | 240.731 | 4.093 | | < 0.001 | |
| Temperature (24°C) * Population (Wyoming) | 0.722 | 0.297 | 242.113 | 2.434 | | 0.016 | |

**Table S10.** Best-fit generalized linear mixed model explaining the proportion of orange in the forewings of the adult moths in the *Amata nigriceps* rearing experiment (n = 252). Explanatory variables in the best-fit model included rearing temperature (24/28°C), population (Macquarie/Wyoming/Mt Tomaree) and sex, and family as a random effect (variance = 0.00024). Intercept gives the estimate for females from Macquarie population that were reared at 20°C. P-values and degrees of freedom were estimated using Satterthwaite’s approximation (*lmerTest* function in R).

| **Terms in the model** | **Estimate** | **SE** | **df** | **t** | **P** |
| --- | --- | --- | --- | --- | --- |
| Intercept | 0.207 | 0.012 | 12.956 | 17.669 | < 0.001 |
| Temperature (24°C) | -0.001 | 0.002 | 238.441 | -0.485 | 0.628 |
| Population (Mt Tomaree) | 0.033 | 0.014 | 12.147 | 2.322 | 0.038 |
| Population (Wyoming) | 0.009 | 0.013 | 12.482 | 0.708 | 0.492 |
| Sex (male) | -0.043 | 0.002 | 239.375 | -17.381 | < 0.001 |

**3. Rearing experiment: genetic basis of warning signal expression**

**a) General linear mixed model approach (i.e., the ‘animal model’ approach)**

**Model:**

$B ~ mu SEX POP TEMP G1 SEX.POP SEX.TEMP SEX.G1 POP.TEMP POP.G1 !r SEX.nrm(ANIM)

**Random variance components:**

|  | (co)Variance component |  |  | **ESTIMATE** | **SE** |
| --- | --- | --- | --- | --- | --- |
| 1 | Residual (Female) |  |  | 0.452 | 1.027 |
| 2 | Residual (Male) |  |  | 2.201 | 0.812 |
| 3 | Genetic correlation | R | 1 | 0.890 | 0.130 |
| 3 | Genetic (Female) | V | 1 | 5.116 | 1.953 |
| 4 | Genetic (Male) | V | 2 | 3.346 | 1.354 |
| 5 | Phenotypic (Female) |  | 1 | 5.568 | 1.074 |
| 6 | Phenotypic (Male) |  | 2 | 5.546 | 0.842 |
|  |  |  |  |  |  |

**Fixed effects:**

|  | **Source** | **NumDF** | **DenDF** | **F-inc** | **F-con** | **M** | **P** |
| --- | --- | --- | --- | --- | --- | --- | --- |
| 18 | mu | 1 | 22.80 | 2524.13 | 2524.13 | . | <.001 |
| 4 | SEX | 1 | 8.90 | 162.90 | 161.90 | A | <.001 |
| 8 | POP | 2 | 21.80 | 4.85 | 4.89 | A | 0.018 |
| 10 | TEMP | 1 | 253.30 | 0.34 | 0.21 | A | 0.649 |
| 5 | G1 | 1 | 29.60 | 0.01 | 0.00 | A | 0.968 |
| 19 | SEX.POP | 2 | 8.50 | 0.01 | 0.00 | B | 0.998 |
| 20 | SEX.TEMP | 1 | 258.80 | 1.10 | 2.17 | B | 0.142 |
| 21 | SEX.G1 | 1 | 169.00 | 4.45 | 4.71 | B | 0.031 |
| 22 | POP.TEMP | 2 | 252.80 | 2.27 | 2.01 | B | 0.136 |
| 23 | POP.G1 | 2 | 31.30 | 0.00 | 0.00 | B | 1.000 |
|  |  |  |  |  |  |  |  |

**Model testing:**

|  | LL | 2LL | d.f. | P |
| --- | --- | --- | --- | --- |
| Full model no constraints | -330.979 |  |  |  |
| Residual variances equal | -332.15 | 2.342 | 1 | 0.125928 |
| Genetic variances equal | -332.316 | 0.332 | 1 | 0.564484 |
| Genetic correlation = 1.0 | -332.867 | 1.102 | 1 | 0.293828 |
| Residual variances = 0.0 | -342.37 | 19.006 | 1 | < 0.001 |
| Genetic variances = 0.0 | -373.78 | 62.82 | 1 | < 0.001 |

**Final model:**

|  | (co)Variance component |  |  | **ESTIMATE** | **SE** |
| --- | --- | --- | --- | --- | --- |
| 1 | Residual |  |  | 1.660 | 0.654 |
| 3 | Genetic correlation | R | 1 | 0.999 | 0.000 |
| 4 | Genetic (both sexes) | V | 2 | 3.827 | 1.259 |
| 5 | Phenotypic (both sexes) |  | 1 | 5.487 | 0.749 |
|  |  |  |  |  |  |

**b) Parent – offspring regression approach**

**Mid-parent – Offspring Regression:**

ANOVA of regression: F_1,7_ = 18.2, P < 0.005

Multiple R = 0.850

R^2^ = 0.722

R^2^_adj_ = 0.683

|  | **β** | **s.e. β** | **B** | **s.e. B** | **t** | **P** |
| --- | --- | --- | --- | --- | --- | --- |
| Intercept |  |  | 0.0510 | 0.0336 | 1.516637 | 0.173142 |
| Mid-parent | 0.84983 | 0.199209 | **0.7263** | **0.1702** | 4.266019 | 0.003719 |

**Mid-parent – Daughter Regression:**

ANOVA of regression: F_1,8_ = 19.6, P < 0.005

Multiple R = 0.842

R^2^ = 0.710

R^2^_adj_ = 0.674

|  | **β** | **s.e. β** | **B** | **s.e. B** | **t** | **P** |
| --- | --- | --- | --- | --- | --- | --- |
| Intercept |  |  | 0.0770 | 0.0316 | 2.438305 | 0.040672 |
| Mid-parent | 0.842502 | 0.190457 | **0.7094** | **0.1604** | 4.423578 | 0.002216 |

**Mid-parent – Son Regression:**

ANOVA of regression: F_1,8_ = 10.7, P < 0.05

Multiple R = 0.757

R^2^ = 0.573

R^2^_adj_ = 0.520

|  | **β** | **s.e. β** | **B** | **s.e. B** | **t** | **P** |
| --- | --- | --- | --- | --- | --- | --- |
| Intercept |  |  | 0.0362 | 0.0422 | 0.857573 | 0.416079 |
| Mid-parent | 0.75706 | 0.230992 | **0.7047** | **0.2150** | 3.277424 | 0.011231 |
